# Free kick instead of cross-validation in maximum-likelihood refinement of macromolecular crystal structures

**DOI:** 10.1107/S1399004714021336

**Published:** 2014-11-22

**Authors:** Jure Pražnikar, Dušan Turk

**Affiliations:** a Department of Biochemistry and Molecular and Structural Biology, Institute Jožef Stefan, Jamova 39, 1000 Ljubljana, Slovenia; b Faculty of Mathematics, Natural Sciences and Information Technologies, University of Primorska, Slovenia; c Center of Excellence for Integrated Approaches in Chemistry and Biology of Proteins, Slovenia

**Keywords:** free-kick refinement, *R*_free_, maximum likelihood, cross-validation

## Abstract

The refinement of a molecular model is a computational procedure by which the atomic model is fitted to the diffraction data. The commonly used target in the refinement of macromolecular structures is the maximum-likelihood (ML) function, which relies on the assessment of model errors. The current ML functions rely on cross-validation. They utilize phase-error estimates that are calculated from a small fraction of diffraction data, called the test set, that are not used to fit the model. An approach has been developed that uses the work set to calculate the phase-error estimates in the ML refinement from simulating the model errors *via* the random displacement of atomic coordinates. It is called ML free-kick refinement as it uses the ML formulation of the target function and is based on the idea of freeing the model from the model bias imposed by the chemical energy restraints used in refinement. This approach for the calculation of error estimates is superior to the cross-validation approach: it reduces the phase error and increases the accuracy of molecular models, is more robust, provides clearer maps and may use a smaller portion of data for the test set for the calculation of *R*_free_ or may leave it out completely.

## Abstract 2

The maximum-likelihood free-kick target, which calculates model error estimates from the work set and a randomly displaced model, proved superior in the accuracy and consistency of refinement of crystal structures compared with the maximum-likelihood cross-validation target, which calculates error estimates from the test set and the unperturbed model.

## 1. Introduction   

Structural biology has immensely impacted our understanding of biological processes by providing insight into molecular structures at the atomic level. The oldest and most widely used approach, which has delivered the majority of structural data to date, is macromolecular crystallography. After diffraction data have been measured and the phase problem has been solved, structural models are built, rebuilt and refined to best interpret the measured data. Every macromolecular model from the last few decades has been subjected to crystallo­graphic refinement, in which the positions of individual atoms are fitted to experimental observations to make the model represent the data in the best way possible. The least-squares (LSQ) formulation of the target function was initially used for refinement in reciprocal space (Sussman *et al.*, 1977 ▶; Hendrickson & Konnert, 1980 ▶). In the 1980s, an initial attempt to use the maximum-likelihood (ML) target function (Lunin & Urzhumtsev, 1984 ▶) was unsuccessful because the phase-error estimates were too low. The success of the ML target was made possible by cross-validation in the form of the *R*_free_ factor (Brünger, 1992 ▶), which was introduced to monitor overfitting (Brünger, 1993 ▶; Kleywegt & Brünger, 1996 ▶). Use of the partial free data concept (Lunin & Skovoroda, 1995 ▶) showed that the refinement was successful. As a consequence, ML refinement has been widely adopted by the crystallo­graphic community (Pannu & Read, 1996 ▶; Bricogne & Irwin, 1996 ▶; Murshudov *et al.*, 1997 ▶; Adams *et al.*, 1997 ▶; Pannu *et al.*, 1998 ▶). Today, the free, model-unbiased fraction of data typically consists of 5% of the diffraction data.

In contrast to the current refinement practice using the ML target, where the model structure factors are compared against the free part of the data, we decided to do the opposite: to free the model by a kick and use the work set of data to calculate phase-error estimates. This approach was inspired by the previous uses of kicks. Kicking is a mathematical procedure which modifies coordinates or *B* factors by a random shift. Kicking is routinely used in model rebuilding and refinement (displacing initial coordinates and *B* values) and in map calculations (Turk, 2013 ▶). We have previously presented the idea that the assessment of phase errors modelled by kicking result in maps of improved quality, termed averaged kick maps (Pražnikar *et al.*, 2009 ▶). We now extend this concept for use in calculation of the target function in refinement by adding a procedure which calculates the model error estimates from a randomly displaced model to the computer program *MAIN* (Turk, 2013 ▶). Hence, the use of kicking in refinement is now dual: firstly as part of coordinate and *B*-factor manipulation to help the minimizer avoid local minima and secondly in the calculation of model error estimates. The two kicking procedures do not interfere with each other. The coordinate manipulation happens before the model enters minimization, whereas the second procedure uses the model in refinement but does not alter it; it only affects the calculation of the coefficients of the target function.

The problem in refinement that we address here is the independence of calculated structure-factor magnitudes (SFMs), often referred to as model ‘bias’. In ML refinement supposedly independent SFMs are used to derive the ‘shape’ of an approximation for the likelihood function; unknown parameters of the likelihood function (σ_A_ or α/β) are determined based on the set of experimental measurements. After the likelihood function has been fully determined in shape and parameters, it can be applied to estimate coordinate or phase errors or as a target in refinement. If we run unrestrained refinement against the work reflections only, then the work SFMs are not independent and produce poor estimates of the parameters of the likelihood function. Nevertheless, the SFMs of the test reflections are to some extent independent and produce more realistic estimates of the parameters. When refinement is restrained by the stereochemical relations, then the SFMs of the test reflections become dependent too, although to a lesser extent than the work reflections. Kicks, however, break the coordinate dependence and the dependence on SFMs and allow the use of all reflections to determine reliable likelihood-function parameters. The consequence of the kick is a model that is a little worse but which is better evaluated. At the same time the model before the kick is better, but its evaluation is less accurate. To overcome the problem, we derive the α and β parameters of the likelihood function from the kicked model and apply them to the model before the kick.

## 2. Methods   

### 2.1. Free-kick refinement   

Firstly, the coordinate error is estimated by comparing the structure factors calculated from the unperturbed model against the working set of observed structure factors. The model is then freed by a kick of the size of the coordinate error estimate, and structure factors from this kicked model are calculated. These free-kick structure factors are then compared with the observed structure factors of the working set and used to calculate the α and β coefficients for the ML function. These coefficients are then applied to the structure factors of the unperturbed model to calculate the forces on atoms during refinement. We call this procedure ML free-kick refinement (ML FK), as it combines the ML formalism with an atomic kick, which frees the atoms from their restraints imposed by the energy function.

To validate the ML FK target, we compared the phases, coordinates and electron-density maps of refined structures with those from the true reference models. The model was refined using the standard cross-validated ML target function (ML CV), ML whiteout cross-validation (ML noCV) and ML FK, which use the test portion of the data, all data and the free-kick structure factors of the work set to estimate the phase errors, respectively. Three sets of comparisons were performed. The first comparison addressed the accuracy of a partial structure refined at a truncated resolution, the second addressed the model bias of a partially incorrectly refined model and the third comparison addressed the robustness of the target based on an example of a group of molecular-replacement solutions with either identical sequences that partially differed structurally or that were identical in fold but different in sequence from the crystal structure.

### 2.2. Refinement protocol   

A macromolecular refinement was obtained through the maximum-likelihood formulation, in which the residual represents the negative logarithm of the likelihood (Lunin *et al.*, 2002 ▶). The residual is represented as
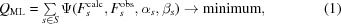
with
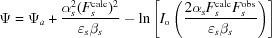
for acentric reflections and 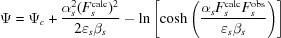
for centric reflections.

Here, *F* ^calc^ and *F* ^obs^ represent the calculated and measured structure factors, respectively. The calculated structure factors include bulk-solvent correction, whereas the *F* ^obs^ are modified by the overall anisotropic *B* correction. The notation *I*_o_ represents the modified Bessel functions and the parameters α and β define the expected phase errors as described in the literature (Lunin & Urzhumtsev, 1984 ▶; Read, 1986 ▶; Lunin & Skovoroda, 1995 ▶; Pannu & Read, 1996 ▶). The *MAIN* algorithm is an implementation of the formulation described by Lunin & Skovoroda (1995 ▶).

Refinement calculations were performed with the crystallographic program *MAIN* (Turk, 2013 ▶) using the same ML target function with three different sets of parameters: the standard ML approach with cross-validation (ML CV), the ML approach by estimating model errors from the working set (ML without cross-validation; ML noCV) and the ML FK approach. To facilitate this calculation, the ML free-kick (ML FK) target function was implemented using the already existing ML target function (Lunin & Skovoroda, 1995 ▶). The only change required was to use the coordinate error estimates calculated from the nonperturbed model and to apply this value as the maximum kick size in the additional calculation of the structure-factor set from the kicked coordinates. This kicked structure-factor set was then used to calculate the α and β coefficients for the ML target function used in derivative map calculation.

The refinement protocol was the same in all cases: 12 cycles (12 × 60 steps) of conjugate-gradient minimization of coordinates were combined with eight cycles (8 × 30 steps) of minimization of *B* factors. In each cycle of positional refinement, the initial coordinate maximum kick size was set to 0.5 Å. The kick was lowered in each cycle until the smallest kick of 0.01 Å was reached. *B*-factor refinement began with uniform *B* factors of 25 Å^2^ followed by *B*-factor kicks. The initial maximal *B*-factor kick was set to 15 Å^2^ and was consecutively lowered until a final kick of 2 Å^2^ was reached. In each cycle, the random seed was increased by one to assure a different randomization of the coordinate and *B*-factor changes in each refinement cycle. In the ML CV approach, the test set of reflections was used for cross-validation, whereas it was only used to monitor refinement in the other approaches. Table 1 ▶ contains information on the total number of reflections used in the calculations and indicates the numbers of reflections used in the various fractions of the test sets.

### 2.3. Molecular-replacement test cases and electron-density map calculation   

To address the convergence and robustness of refinement, we chose five starting cases of molecular-replacement solutions of the crystal structures of cathepsin H (PDB entry 8pch; Gunčar *et al.*, 1998 ▶), ammodytin L (PDB entry 3dih; D. Turk, G. Gunčar & I. Krizaj, unpublished work), the stefin B tetramer (PDB entry 2oct; Jenko Kokalj *et al.*, 2007 ▶), cherry allergen (PDB entry 2ahn; Y. Dall’Antonia, T. Pavkov, H. Fuchs, H. Breiteneder & W. Keller, unpublished work) and the choline acetyltransferase–choline complex (PDB entry 2fy2; Kim *et al.*, 2006 ▶). The structures of actinidin (Baker, 1980 ▶), *Crotalus atrox* phospholipase A_2_ (Keith *et al.*, 1981 ▶), stefin B (Stubbs *et al.*, 1990 ▶), thaumatin (Ko *et al.*, 1994 ▶) and choline acetyltransferase (Cai *et al.*, 2004 ▶) were used as the search models (Pražnikar *et al.*, 2009 ▶). Refinement was performed using four different sizes of the test set: 1, 2, 5 and 10%. For each test-set size, 31 different test sets of reflections were randomly selected. To produce a unique test set, each was generated with a different random seed. Table 1 ▶ shows the number of test reflections per shell for different sizes of the test sets. Refinement was performed using five resolution shells with data truncated to 3 Å resolution.

Electron-density maps were calculated in *MAIN* (Turk, 2013 ▶) using the ML CV and ML FK approaches to calculate the phase-error estimates and the corresponding α and β coefficients used in the σ_A_-weighted map calculation (Read, 1986 ▶).

The real-space *R* factor along the chain was calculated in *MAIN* according to the procedure described by Kleywegt & Jones (1995 ▶).

## 3. Results   

### 3.1. Assessment of the accuracy of refinement on truncating the resolution of the data   

**Figure 1:**
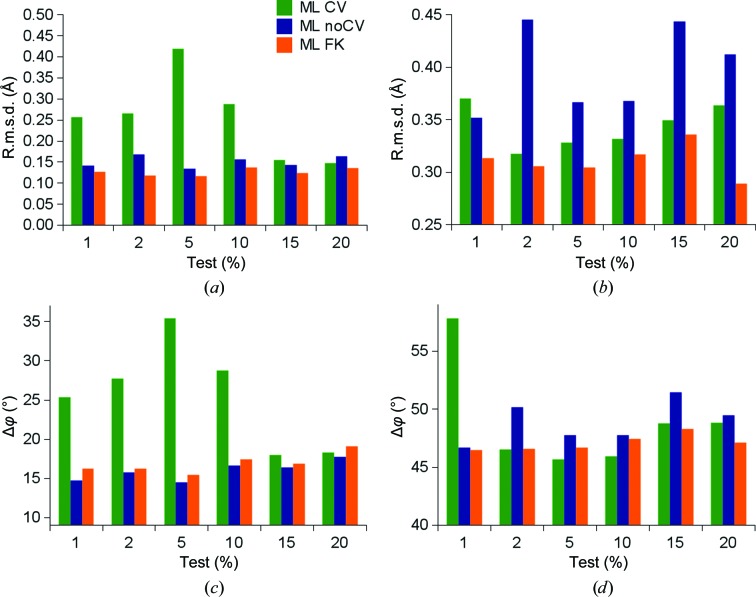
Accuracy of refinement. Coordinate and phase errors of the crambin structure refined at truncated resolution. ML CV, ML noCV and ML FK refinements at 2.0 Å resolution were performed using 1, 2, 5, 10, 15 and 20% test sets. (*a*) Root-mean-square distance (r.m.s.d.) of C^α^ atoms of the partial crambin structure against the deposited structure of crambin. (*b*) R.m.s.d. of C^α^ atoms of the polylalanine model against the deposited structure of crambin. (*c*) Phase error of partial crambin structure against the deposited structure of crambin. (*d*) Phase error of the polyalanine model against the deposited structure of crambin.

**Figure 2:**
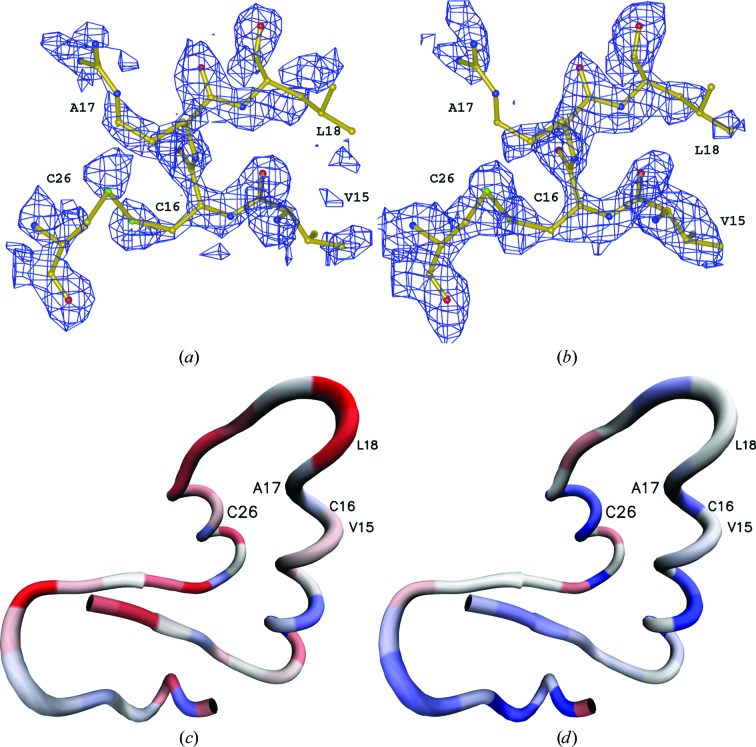
Electron-density analysis of crambin refined at truncated resolution. The 2*mF*_o_ − *DF*_c_ electron density at the 1.0σ contour level of polyalanine models around residues Val15, Cys15, Arg17, Lys18 and Cys26 of the deposited structure of crambin. The map *R* factor along the crambin chain was calculated residue-by-residue between the *F*_c_ map of the final deposited model and the 2*mF*_o_ − *DF*_c_ map of the refined polyalanine model. The electron-density *R* factor ranges from 0.28 (blue) to 0.67 (red). (*a*) ML CV electron-density map using a 5% test set. (*b*) ML FK electron-density map using a 1% test set. (*c*) Residue-by-residue map *R* factor of ML using a 5% test set. (*d*) Residue-by-residue map *R* factor of ML FK using a 1% test set.

To analyze the accuracy of refinement of the three target functions, we compared the displacement of C^α^ atoms of the refined structures with those of the true structure. For this example, we chose the crambin structure PDB entry 1ejg (Jelsch *et al.*, 2000 ▶) refined at a resolution of 0.54 Å, which makes it the macromolecule with the highest resolution in the entire PDB. The crambin amino-acid chain (Figs. 1 ▶ *a* and 1 ▶ *c*) and its polyalanine (Figs. 1 ▶ *b* and 1 ▶ *d*) model were refined using the ML CV, ML noCV and ML FK target functions against data truncated to 2.0 Å resolution with different fractions of test data. An overview of the coordinate (Figs. 1 ▶ *a* and 1 ▶ *b*) and phase (Figs. 1 ▶ *c* and 1 ▶ *d*) errors demonstrates that the ML CV target function strongly depends on the size of the test portion of data and that the lowest deviations from the reference structure are exhibited by the structures refined using the ML FK target. Coordinate errors were calculated by the root-mean-square distance (r.m.s.d.), whereas the phase errors were calculated by comparing the structure factors from the reference structure with the refined models. Among the structures refined using the ML CV target the smallest coordinate and phase errors were provided when the test portion contained at least 15% of the data (Figs. 1 ▶ *a* and 1 ▶ *c*). In contrast, the ML FK target does not exhibit such a strong test-set size dependence. When the whole crambin model (Fig. 1 ▶ *a*) was tested, the ML FK refinement yielded an r.m.s.d. on C^α^ atoms of between 0.12 and 0.14 Å, whereas the deviations of the ML CV target ranged from 0.15 to 0.42 Å. For the polyalanine model, all target functions behaved worse than for the correct poly­peptide sequence (Figs. 1 ▶ *b* and 1 ▶ *d*). The ML FK refinements yielded an r.m.s.d. that ranged between 0.29 and 0.34 Å and phase errors that ranged from 47 to 48°. With the ML CV target, the refined structures yielded r.m.s.d.s from 0.32 to 0.36 Å and the phase errors ranged from 46 to 58°. Additionally, refinement of the polyalanine model shows the highest deviations for the ML noCV target. Interestingly, in this experiment the lowest coordinate error does not fully coincide with the lowest phase error; nevertheless, we felt that we should use the phase error in further analysis owing to its widespread use. To make the numerical analysis understandable in terms of the three-dimensional structure, two σ_A_-weighted electron-density maps were calculated with the polyalanine model around residues Val15, Cys15, Arg17, Leu18 and Cys26 and were displayed on the background of the deposited structure of crambin (Figs. 2 ▶ *a* and 2 ▶ *b*). The chain trace of crambin is shown with the colour-coded real-space *R* factor of the maps (Figs. 2 ▶ *c* and 2 ▶ *d*). Evidently, the maps resulting from ML FK refinement and ML FK phase-error estimates for the weights of the structure factors in the maps are better connected, less noisy and have a lower real-space *R* factor, as indicated by the blue shift of Fig. 2 ▶(*d*) in comparison to Fig. 2 ▶(*c*).

### 3.2. Assessment of model bias from a partially incorrect model   

**Figure 3:**
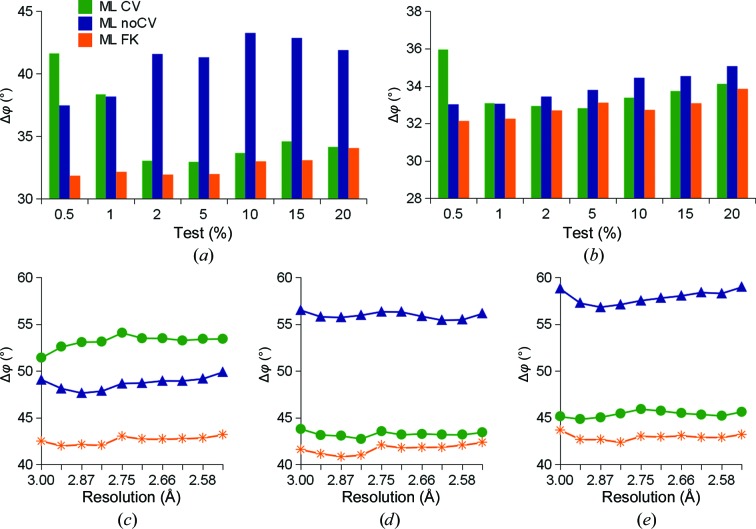
Phase-error comparisons of the refined partially wrong model. The final phase errors for PDB entry 1zen with misplaced residues and the new correct model are shown. ML CV, ML noCV and ML FK refinements were performed using 0.5, 1, 2, 5, 10, 15 and 20% test sets. The phase error in the higher resolution shells was calculated in ten resolution shells between 3.0 and 2.5 Å. (*a*) Phase errors of models refined at 3.0 Å resolution. (*b*) Phase errors of models refined at 2.5 Å resolution. (*c*–*e*) Phase error of a 3.0 Å resolution refined structure in higher resolution shells (*c*) using a 1% test set, (*d*) using a 5% test set and (*e*) using a 10% test set.

To address the sensitivity to model bias, we employed PDB entry 1zen, which has previously been used for this purpose (Terwilliger *et al.*, 2008 ▶; Pražnikar *et al.*, 2009 ▶). The 1zen structure contains over 10% of residues misplaced from the positions observed in the closely related PDB entry 1b57. To create the reference, the 1zen model was manually rebuilt and re-refined using the traditional approach of the ML CV target function. The deposited model with solvent and heteroatoms excluded was then subjected to refinement at resolutions of 2.5 and 3.0 Å using the three target functions and test portions including 0.5, 1, 2, 5, 10, 15 and 20% of the measured data. In this comparison, ML FK evidently yielded the lowest phase errors and the lowest test-set dependence among the targets in this comparison (Figs. 3 ▶ *a* and 3 ▶ *b*). Furthermore, model bias was also analyzed in a region of data not used in the refinement. To this end, we used the models refined at 3.0 Å resolution. We calculated the structure factors to 2.5 Å resolution and compared the phase errors in ten resolution shells in the interval between 3.0 and 2.5 Å (Figs. 3 ▶ *c*, 3 ▶ *d* and 3 ▶ *e*). Again, the ML FK target performed best in all resolution shells, while ML CV and also ML noCV showed a strong dependence on the size of the test portion of data. Furthermore, ML FK yielded the lowest phase errors among the compared targets in an overall comparison.

### 3.3. Assessment of convergence and robustness from molecular-replacement solutions   

**Figure 4:**
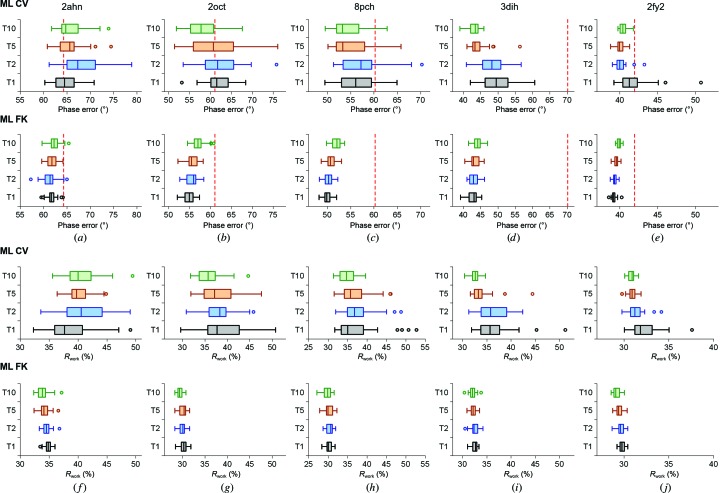
Distribution of phase errors and of *R*_work_. The graphs show the distribution of phase errors and of *R*_work_ after refinement at 3.0 Å resolution for 31 different test sets. Red dashed lines show the starting phase error of the model. ML CV (*a*–*e*) and ML FK (*f*–*j*) refinement target functions were used. The test-set sizes are 1, 2, 5 and 10%. On the graphs they are denoted T1, T2, T5 and T10, respectively. The cases used are cherry allergen (PDB entry 2ahn) (*a*, *f*), stefin B tetramer (2oct) (*b*, *g*), cathepsin H (8pch) (*c*, *h*), ammodytin L (3dih) (*d*, *i*) and choline acetyltransferase (2fy2) (*e*, *j*).

To analyze the robustness and convergence of the target functions in refinement, we chose five cases starting with molecular-replacement solutions. Analysis of the phase errors of the refined molecular-replacement models show that the phase errors and variability of structures refined with the ML FK approach are lower in all cases (Fig. 4 ▶). Fig. 4 ▶ also reveals the general trend of the ML FK function: the size of the work set negatively correlates with the phase error. This relationship is not evident for the ML CV approach, where a 10% size of the test set resulted in the lowest phase error in one instance (Fig. 4 ▶ *d*). Concerning the distribution of the final phase errors, the small size of the test set, on which the scaling of the ML CV approach depends, evidently produces much variation. Comparison of Fig. 4 ▶ with Table 1 ▶ indicates that the spread of phase errors is larger with fewer data in the test set in the ML CV approach. This comparison also makes evident that the spreads of the phase errors of the largest test sets (10% of the data) of the ML CV cases are notably larger than for the ML FK cases. The narrowest spread of phase errors for the 2fy2 case with the largest test-set sizes also reflects the fact that in this case the starting molecular-replacement model was most similar in structure and sequence to the final structure.

**Figure 5:**
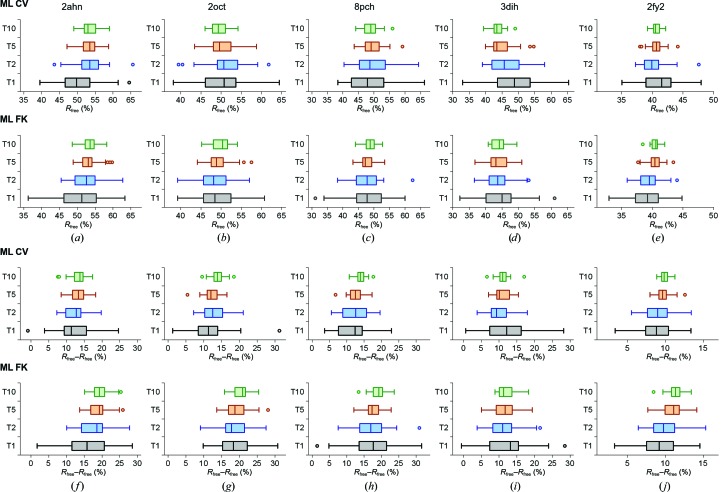
Distribution of *R*_free_ and of the difference between *R*_free_ and *R*_work_. The graphs show the distribution of *R*_free_ and of the final difference between *R*_free_ and *R*_work_ after refinement for 31 different test sets containing 1, 2, 5 and 10% of the data. ML CV (*a*–*e*) and ML FK (*f*–*j*) refinement target functions were used at 3.0 Å resolution. On the graphs they are denoted T1, T2, T5 and T10, respectively. The cases used are cherry allergen (PDB entry 2ahn) (*a*, *f*), stefin B tetramer (2oct) (*b*, *g*), cathepsin H (8pch) (*c*, *h*), ammodytin L (3dih) (*d*, *i*) and choline acetyltransferase (2fy2) (*e*, *j*).

**Figure 6:**
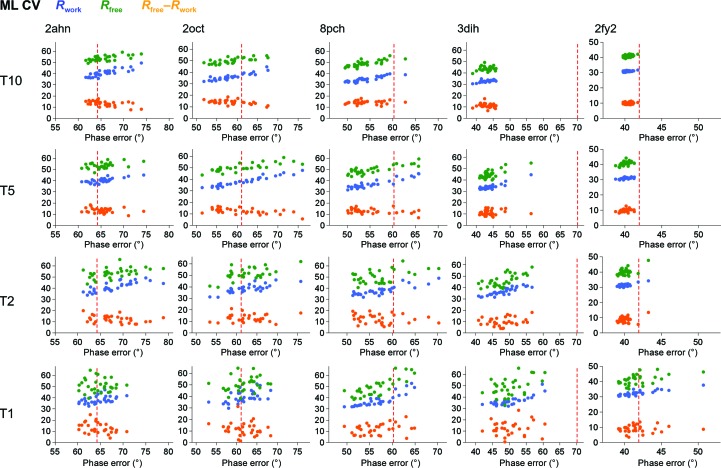
*R* factors against phase errors for ML CV. The cases used in the columns from left to right are cherry allergen (PDB entry 2ahn), stefin B tetramer (2oct), cathepsin H (8pch), ammodytin L (3dih) and choline acetyltransferase (2fy2). The test-set sizes are 1, 2, 5 and 10%, indicated by T1, T2, T5 and T10, respectively. Red dashed lines show the starting phase error. *R*_work_, *R*_free_ and *R*_free_–*R*_work_ are plotted as dots for each of the 31 cases on the vertical axes in blue, green and orange, respectively.

The *R*_free_ value distribution (Fig. 5 ▶) appears to be related to the size of the test-set portion, irrespective of the ML approach used. The *R*_free_ value distribution is tightest at 10% of the data and widest at 1%. This analysis indicates that the use of a larger test-set size to calculate *R*_free_ stabilizes the calculation and is less prone to accidental choice of the data in the test set. However, the increase in the test-set size has undesirable consequences: it decreases the work set, which consequently lowers the number of reflections used in the refinement procedure and thereby increases the phase error. A similar relationship holds for the *R*_free_–*R*_work_ difference (Fig. 5 ▶). In contrast, the *R*_work_ distributions (Fig. 4 ▶) evidently show less variability for the ML FK approach than for the ML CV approach. Clearly, the wider distribution of *R*_work_ for the ML CV approach is a consequence of the small amount of data used in the test set (small sample size) and is therefore more sensitive to their accidental choice. The opposite is true for the ML FK approach, where most of the data used to calculate the α and β coefficients of the ML function are the same. This analysis indicates that the data set included in the test set is underrepresented to enable robust and convergent refinement at all stages of structure determination. In particular, this approach increases the spread of possible solutions at large phase errors. This relationship is also reflected in the trends on comparison of the phase errors (Fig. 4 ▶) and *R*_work_ (Fig. 6 ▶): the decrease in the *R*_work_ values in the ML FK approach reflects the decrease of the size of the work set, while this trend is not pronounced in the *R*_work_ plots for the ML CV approach. This analysis indicates that *R*_work_ can be lowered when a smaller number of reflections are fitted, whereas the use of a smaller number of reflections in refinement also results in models that deviate more from their true target.

**Figure 8:**
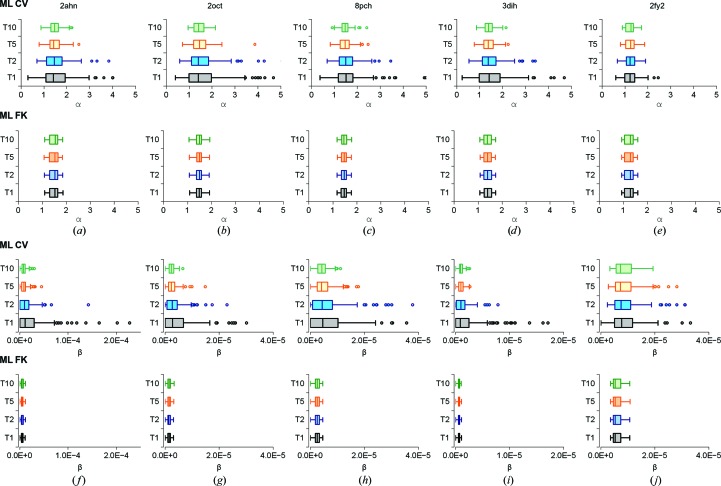
Distribution of the parameters α and β for molecular-replacement solutions at 3.0 Å resolution for 124 different test sets (31 for each test-set size). The cases used in the columns from left to right are cherry allergen (PDB entry 2ahn) (*a*, *f*), stefin B tetramer (2oct) (*b*, *g*), cathepsin H (8pch) (*c*, *h*), ammodytin L (3dih) (*d*, *i*) and choline acetyltransferase (2fy2) (*e*, *j*).

To provide a further insight into the robustness and convergence of the ML CV and ML FK refinement approaches, two-dimensional plots of the phase errors and *R* factors of the refined models are displayed in Figs. 6 ▶ and 7 ▶. Comparison between ML CV and ML FK reveals a tighter clustering of phase errors and *R* values for the ML FK approach. Combining this analysis with the lower phase-error analysis shown above (Fig. 4 ▶) demonstrates that the ML FK approach yields more robust and convergent refinement results than the ML CV approach currently in use. The ML FK target exhibited better convergence and accuracy compared with the currently used ML CV target in these tests. Furthermore, the analysis shows that this difference is a consequence of the phase-error estimate procedure, which relies on the statistics of the work set instead of the test set. To show that this is a direct consequence of the different estimation of parameters by the two ML approaches in Fig. 8 ▶, estimates of α and β for all of molecular-replacement solutions prior to refinement are shown. The figure reveals that the distribution of α and β estimates is notably wider for the ML CV function in comparison with the estimates calculated using ML FK and that the differences in spread are not confined to cases with lower amounts of reflections in the test set.

## 4. Discussion   

The presented analysis demonstrates that the use of the ML FK target in refinement will deliver more accurate structures that correspond to the experimental data better than the currently most often used ML CV target function. The ML FK target thus appears to depend less on model bias and is less prone to outliers that result from the selected test-set reflections. This behaviour can be explained in two ways. The first is the difference in the concept, as explained in §[Sec sec1]1. We reconsidered the assumption that the coordinate errors of the model are random which underlies the ML approach and realised that this assumption is not entirely considered in the ML CV approach because the test-set SFMs are also biased by the model. We compensated for this model bias with a simulation in which the model atoms were randomly displaced by an appropriate kick. The second explanation involves the considerable differences in the sizes of the data sets used to calculate the coordinate error estimates. ML FK uses the work set (95% or more of the measured data), whereas the ML CV function typically relies on the test set (5% of measured data), which makes ML FK estimates more accurate and more independent of random variation in the test set compared with ML CV estimates.

The motivation for this work was that excluding data from refinement introduces bias from their absence into the structure, yet the introduction of all data should not be at the expense of the accuracy of the structure. The elementary criterion for assessment of the success of refinement is the convergence of the atomic model towards the true structure. The closer that refinement brings the model to the true structure the more accurate it is. The ML CV approach proved to be more convergent than the LSQ function (Pannu & Read, 1996 ▶). This work suggests that one can achieve even better convergence towards the true structure by the use of the ML FK approach with larger work sets. Hence, the overfitting of models by the ML FK approach is smaller than that by the ML CV approach.

**Figure 9:**
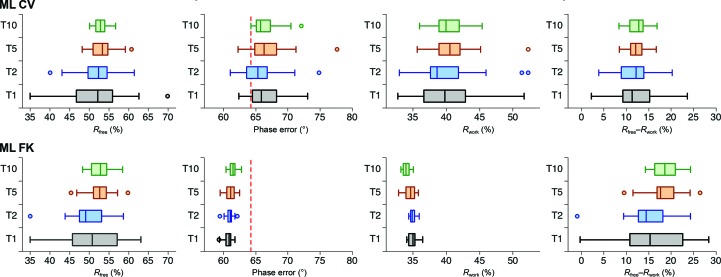
The spread of *R*_free_, phase error, *R*_work_ and *R*_free_−*R*_work_ for a reversely built structure. The 2ahn structure was built into electron density in the reverse direction and refined by the ML CV and ML FK approaches using 10, 5, 2 and 1% test sets, denoted T10, T5, T2 and T1, respectively.

The presented work also suggests that the uses of *R*_free_ may be reconsidered. The first use of partial data in refinement was described by Silva & Rossmann (1985 ▶), where they reduced the size of a data set of approximately 300 000 measured reflections to overcome the computational limitations of an at the time enormous data set from *Southern bean mosaic virus* by exploiting the strict tenfold icosahedral noncrystallographic symmetry. They used a smaller part of the data (1/7) for the work set and the larger part of the data (6/7) for the test set and showed that the structure can be successfully refined against a smaller part of the data in the case where noncrystallographic symmetry allowed the reduction of the reciprocal-space asymmetric unit. Later, in contrast to this use, the measured data were split into a large part for the work set and a small part for the test set for validation purposes. Two main roles for the use of *R*_free_ emerged: the detection of wrong structures and the prevention of overfitting. To address the use of *R*_free_ as indicator of wrong structures, we repeated the Kleywegt and Jones experiment (Kleywegt & Jones, 1995 ▶; Kleywegt & Jones, 1997 ▶) and built the 2ahn structure in the reverse direction and then refined it in the absence of solvent using the ML CV and ML FK approaches. Fig. 9 ▶ shows that *R*_free_ stayed around 50% and *R*_free_–*R*_work_ around 15% in the case of the reverse structure regardless of the ML approach and the fraction of data used in the test set. These values indicate that there is a fundamental problem with the structure, which supports the further use of *R*_free_ as an indicator. However, using the ML FK approach the size of the test set does not matter. It can be as small as 1% of the data or likely even less and the message about a fundamental problem with the structure solution will still be provided. Once it has been established that the structure solution is correct, the test part of the data can be merged with the work part to deliver a structure of higher accuracy. We wish to add that an experienced crystallographer would realise that the structure was built in the wrong direction owing to numerous mismatches of the model and the electron-density maps and inconsistency of the three-dimensional fold with the sequence, and that other validation warnings were also disregarded.

Regarding the use of *R*_free_ to prevent overfitting, we looked back in time to the circumstances in which *R*_free_ was introduced into refinement in 1992 (Brünger, 1992 ▶). In 1993, Brünger wrote that ‘published crystal structures show a large distribution of deviations from ideal geometry’ and that ‘the Engh & Huber parameters allow one to fit the model with surprisingly small deviations from ideal geometry’ (Brünger, 1993 ▶). The work of Engh & Huber (1991 ▶) introduced targets for bond and angle parameters derived from the crystal structures of small molecules in the Cambridge Structural Database (Allen *et al.*, 1987 ▶). Nowadays, statistically derived parameters are routinely used in refinement. Moreover, noting the problem of structural quality, numerous validation tools have been developed and have became an unavoidable part of structure determination and deposition. In refinement the practice has been established that the deviations from ideal geometry are defined as a target used to scale crystallographic energy terms. Hence, the overfitting of models which leads to severe deviations from ideal geometry is no longer really possible. Hand in hand with the progress in tools delivering better models, the amount of data used for the test set has also gradually decreased from an initial 10% or more to 5% or less. Its portion is now practically limited by the request for statistical reliability of the ML CV parameters. To conclude, our understanding is that in the early 1990s in the absence of rigorous geometric restraints structure validation was first introduced in reciprocal space with *R*_free_. Nowadays, however, overfitting can be controlled in real space by the rigorous use of geometric restraints and validation tools. For example, refinement runs restraining the overall r.m.s.d. of bond lengths to 0.01 and 0.005 Å in comparison with the default value of 0.02 Å lead to a decrease in the *R*_free_–*R*_work_ differences with a simultaneous increase in the phase error and *R*_work_ (data not shown). Since the ML FK approach allows the use of all data in refinement with a gain in structure accuracy and thereby delivers lower model bias, this work encourages the use of all data in the refinement of macromolecular structures.

During the development of a procedure which has higher accuracy than ML CV and uses all data in refinement, the ML FK procedure was not the first concept to be tested. Therefore, we anticipate further improvements and simplifications in the future such as the generation of kick structure factors directly from the unperturbed structure factors of the model. Once sufficient experience has been gathered, tabulated values of parameters of ML functions may enter into use. The ML FK approach described here is however simple to implement once the ML refinement code is in place and has a low computational cost, so we expect its broad use.

## Figures and Tables

**Figure 7:**
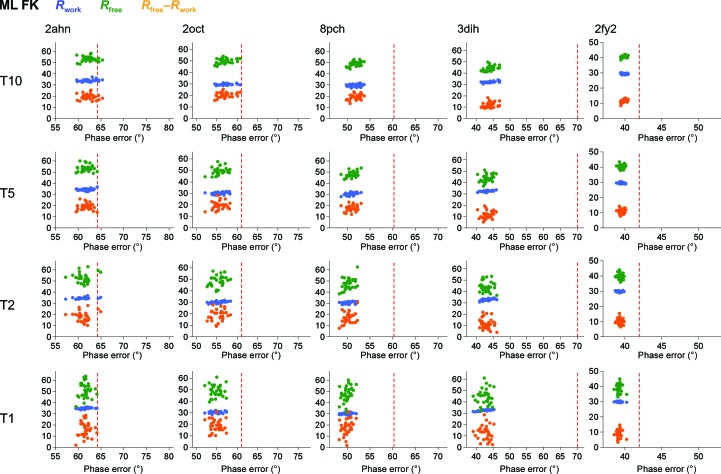
*R* factors against phase errors for ML FK. The cases used in the columns from left to right are cherry allergen (PDB entry 2ahn), stefin B tetramer (2oct), cathepsin H (8pch), ammodytin L (3dih) and choline acetyltransferase (2fy2. The description of the figure is the same as that for Fig. 6 ▶.

**Table 1:** Number of test-set reflections The cases used are crambin (PDB entry 1ejg), class II fructose-1,6-bisphosphate aldolase (1zen), cherry allergen (2ahn), stefin B tetramer (2oct), cathepsin H (8pch), ammodytin L (3dih) and choline acetyltransferase (2fy2). The test-set sizes are 1, 2, 5 and 10%. In the table they are denoted T1, T2, T5 and T10, respectively. The bottom row shows the total number of reflections used in refinement. The crambin data set encompasses the data truncated to 2 resolution, whereas the others encompass the data at 3 resolution.

	1ejg	1zen	2ahn	2oct	8pch	3dih	2fy2
T10	232	1116	412	389	461	347	1419
T5	114	544	209	193	235	174	715
T2	42	209	83	77	93	69	290
T1	16	103	42	39	45	35	143
Total reflections	2397	11334	4158	3920	4603	3508	14205
